# Enhanced TiO_2_/SiC_x_ Active Layer Formed In Situ on Coal Gangue/Ti_3_C_2_ MXene Electrocatalyst as Catalytic Integrated Units for Efficient Li-O_2_ Batteries

**DOI:** 10.3390/nano14030278

**Published:** 2024-01-29

**Authors:** Zhihui Sun, Nan Zhou, Meng Li, Binbin Huo, Kai Zeng

**Affiliations:** 1 School of Mines, China University of Mining and Technology, Xuzhou 221116, China; 5724@cumt.edu.cn (N.Z.);; 2 Institute of Smart City and Intelligent Transportation, Southwest Jiaotong University, Chengdu 610032, China

**Keywords:** mining solid waste, TiO_2_ catalytic units, amorphous/crystalline heterostructure, bifunctional electrocatalysts, Li-O_2_ battery

## Abstract

The pursuit of efficient cathode catalysts to improve cycle stability at ultra-high rates plays an important role in boosting the practical utilization of Li-O_2_ batteries. Featured as industrial solid waste, coal gangue with rich electrochemical active components could be a promising candidate for electrocatalysts. Here, a coal gangue/Ti_3_C_2_ MXene hybrid with a TiO_2_/SiC_x_ active layer is synthesized and applied as a cathode catalyst in Li-O_2_ batteries. The coal gangue/Ti_3_C_2_ MXene hybrid has a tailored amorphous/crystalline heterostructure, enhanced active TiO_2_ termination, and a stable SiC_x_ protective layer; thereby, it achieved an excellent rate stability. The Li-O_2_ battery, assembled with a coal gangue/Ti_3_C_2_ MXene cathode catalyst, was found to obtain a competitive full discharge capacity of 3959 mAh g^−1^ and a considerable long-term endurance of 180 h (up to 175 cycles), with a stable voltage polarization of 1.72 V at 2500 mA g^−1^. Comprehensive characterization measurements (SEM, TEM, XPS, etc.) were applied; an in-depth analysis was conducted to reveal the critical role of TiO_2_/SiC_X_ active units in regulating the micro-chemical constitution and the enhanced synergistic effect between coal gangue and Ti_3_C_2_ MXene. This work could provide considerable insights into the rational design of catalysts derived from solid waste gangue for high-rate Li-O_2_ batteries.

## 1. Introduction

Lithium oxygen batteries (LOBs) with high energy density are hailed as the most promising next-generation high-efficiency energy storage devices [1,2]. However, the kinetics of oxygen reduction/evolution reactions (ORR/OERs) occurring at the cathode of LOBs are particularly sluggish, leading to increased voltage polarization and decreased cycling stability; thus, the further commercial development of LOBs is severely limited [3,4]. At the same time, discharge products (Li_2_O_2_) with low conductivity and poor solubility could accumulate on the surface of the cathode, blocking ion/electron diffusion channels and causing serious degradation in battery performance [5,6]. The rational design of efficient and stable dual-function electrocatalysts which can accelerate the dynamics of ORR/OERs while suppressing the formation of byproducts is key in the pursuit of achieving large-scale applications of LOBs [7]. Carbon-based materials with high conductivity and well-interconnected cellular structures have been wildly verified to be unstable under the nucleophilic attack of O^2-^ and the high overpotential in LOBs [8,9]. Noble-metal-based hybrids which deliver excellent electrocatalytic activity are limited by their high costs [10,11]. Featuring unique magnetic exchange interaction, transition metal oxides/sulfides show good electrocatalytic activities in promoting charge transfers and reducing activation barriers [12,13,14]. Nevertheless, transition metal oxides exhibit narrow catalytic selectivity for ORR/OERs and, in turn, poor electrochemical performance [15,16].

Coal gangue is one of the largest industrial solid wastes discharged during coal mining and washing processing. Coal gangue has low carbon content, and its composition is closely related to the geological conditions and coal mining technologies used in its production. Usually, coal gangue mainly contains SiO_2_ and Al_2_O_3_, as well as small amounts of Fe_2_O_3_, CaO, and MgO, and trace rare elements (Ga, Co, Ti, V) [17]. The composition distribution ratio of coal gangue definitively determines its possible utilization pathways [18]. For example, coal gangue with a silicon aluminum ratio greater than 0.5 can be used to extract aluminum sources or other chemical products. High-carbon coal gangue can be calcined for power generation or processed through physical and chemical processes to form inorganic materials with high electrochemical activities. Characterized by low calorific value and difficult utilization, a large number of coal gangues are stacked on the surface, causing soil erosion, landslide, debris flow, and other geological disasters [19]. In addition, coal gangue can produce many toxic and harmful substances under the effect of infiltration and leaching by rain, which thereby trigger a series of serious ecological pollution problems [20]. Fortunately, coal gangue contains highly active clay minerals such as quartz and kaolinite, which are rich in silicon/aluminum oxides that are connected by interfacial oxygen bridge bonding [21]. The efficient modulation of Si/Al active components is the key to improving the value-added utilization of coal gangue [22]. In situ-formed amorphous SiC_X_/SiO_X_ electrocatalysts with abundant oxygen vacancies derived from coal gangue exhibit excellent electrochemical catalytic activity in LOBs. Furthermore, the synergistic combination of transition metal oxides and stable SiO_2_ synthetized from coal gangue could significantly enhance the adsorption ability of discharge intermediate and in turn optimize the electrochemical reaction path of discharge products during ORRs [23]. However, restricted by the low electric conductivity and poor catalytic selectivity for oxygen redox, coal gangue delivers unsatisfactory electrochemical performance in LOBs at ultra-high rates. 

Transition metal carbides (MXenes) have attracted widespread attention in LOBs to promote the dynamic of OER and ORR [24,25]. MXenes have a distinctive two-dimensional structure and metallic electrical conductivity, which could provide sufficient electrochemical reaction spaces for the formation and decomposition of products and ensure the rapid transfer of charge on the surface [26]. Moreover, there are plenty of functional groups fabricated on the surface of MXenes; these serve as active sites in optimizing the ion diffusion pathway and the adsorption/desorption performance of discharge products; additionally, these encourage their stable interaction in solvent [27]. In detail, the transitional metal oxide protective layer is in situ-prepared on the surface of MXene, leading to an increased number of hydrogen-active sites and enhanced electrochemical activity [28].

Herein, we innovatively fabricated the coal gangue/Ti_3_C_2_ MXene electrocatalyst with an amorphous/crystalline heterointerface; this can potentially be utilized as a cathode electrocatalyst in LOBs. The noncrystalline coal gangue nanoparticles could provide a stable SiC_X_ protective layer and promote the formation of TiO_2_ termination with high electrochemical activity for Ti_3_C_2_ MXene. Simultaneously, Ti_3_C_2_ MXene with a multilayered graphene-like structure can significantly contribute to improving the reaction kinetics and optimizing the product storage of LOBs. The coal gangue/Ti_3_C_2_ MXene electrocatalyst delivers a splendid electrochemical performance, a superior endurance lifespan, and an incredible high-rate capability in LOBs. The detailed characterization analysis reveals that the in situ-formed TiO_2_/SiC_X_ catalytic-integrated units on the surface of the coal gangue/Ti_3_C_2_ MXene hybrid make a crucial difference in improving the electrochemical stability of LOBs at high rates.

## 2. Materials and Methods

### 2.1. Synthesis of Activated Coal Gangue Precursor

The natural coal gangue was poured into a 500 mL zirconia milling pot and cracked into micron-level powder through high-energy ball milling at a rotation of 3000 rpm for five 1 h cycles. The coal gangue powder and Na_2_CO_3_ were weighed according to a mass ratio of 10:6 and ground into a coal gangue powder mixture. Then, the mixture was transferred into a porcelain boat and heated at 900 °C for 3 h. The calcination products were immersed in a H_2_SO_4_ solution for 6 h and then washed and dried, respectively. The dry powder was placed in a H_2_SO_4_ solution (pH 2) for 24 h. Finally, the resulting precipitate was washed with deionized water and ethanol 3~4 times.

### 2.2. Synthesis of Ti_3_C_2_ MXene

The synthesis of Ti_3_C_2_ MXene is referred to in a previous report [29]. A measure of 2 g lithium fluoride (LiF) powder was poured into 40 mL of hydrogen chloride (9 M) solution with continuous magnetic stirring. Then, 2.0 g Ti_3_AlC_2_ MAX powder was transferred slowly into the above solution with magnetic stirring at 40 °C. The whole mixture was sealed into a 100 mL hydrothermal autoclave and maintained at a temperature of 90 °C for 20 h. The remaining precipitate was washed with deionized water and ethanol under high-speed centrifugation 5~6 times until the pH value of the product was adjusted to ~7. Finally, the collected precipitate was dried at 60 °C and calcined at 350 °C for 2 h in an Ar atmosphere to obtain the desired Ti_3_C_2_ MXene.

### 2.3. Synthesis of Coal Gangue@Ti_3_C_2_ MXene

The synthesis of coal gangue@Ti_3_C_2_ MXene is referred to in a previous report [23]. The activated coal gangue precursor and glucose were weighed according to a mass ratio of 4:1 and dissolved in deionized water with continuous magnetic stirring. The prefabricated Ti_3_C_2_ MXene was added into the above dispersion and stirred for 1 h. The obtained homogeneous solution was transferred and sealed into a 100 mL hydrothermal autoclave and maintained at a temperature of 180 °C for 12 h. The resultant mixture was washed with deionized water and ethanol under high-speed centrifugation 5~6 times. Finally, the desired product was calcined at 750 °C for 4 h under an Ar atmosphere to form the coal gangue@Ti_3_C_2_ MXene. 

### 2.4. Materials Characterization

The micro-morphology and elemental distribution were tested using a Zeiss Sigma 300-SEM (Carl Zeiss AG, Oberkochen, Germany) and a Tecnai G20-TEM (FEI, Hillsborough, OR, USA). The crystal structure and chemical composition of the as-prepared catalysts was recorded using an X-ray diffractometer with Cu Kα radiation (XRD, Bede Scientific Ltd., Centennial, CO, USA; Operated at 45 mA and 40 KV; Bruker Corporation, Waltham, MA, USA) and X-ray photoelectron spectroscopy with Al-K radiation (XPS, ESCALAB, 250Xi; Thermo Fisher Scientific, Waltham, MA, USA). 

### 2.5. Cathode Preparation and Measurement

The well-prepared cathode catalysts (coal gangue@Ti_3_C_2_ MXene, Ti_3_C_2_ MXene), conductive additive (acetylene black), and polymer binder (polyvinylidene fluoride, PVDF) were mixed according to a mass ratio of 6:3:1. The whole mixture was poured into a 100 mL zirconia milling pot and cracked through high-energy ball milling at a rotation of 1000 rpm for three 30 min cycles. The ball-milled products were dissolved in N-Methyl pyrrolidone and stirred for 12 h to form a thick and uniform slurry. Then, the prepared slurry was sprayed onto the surface of carbon paper using a spray gun under a high-temperature irradiation area produced by an infrared baking lamp. Finally, the catalyst-based cathode, with a loading mass of ~0.7 mg cm^–2^, was cut into a disk (Φ = 12 mm) and saved in a glovebox.

The 2032 coin-type LOBs were assembled in a glovebox with the as-prepared cathode, lithium anode flakes, a glass microfiber diaphragm (Grade GF/D), and an electrolyte (1.0 M lithium bis (trifluoromethane sulfonyl) dissolved in dimethyl sulfoxide). The assembled LOBs were placed in a self-made sealed glass bottle with oxygen. The electrochemical performance of the LOBs was tested using the Blue Electric Battery Testing System (LANHE CT3001A), Wuhan Blue Electric Company, Wuhan, China. The current densities and specific capacities of the LOBs were normalized through the weight of the cathode catalysts.

## 3. Results

### 3.1. Morphological and Structural Characterization

**Figure 1:**
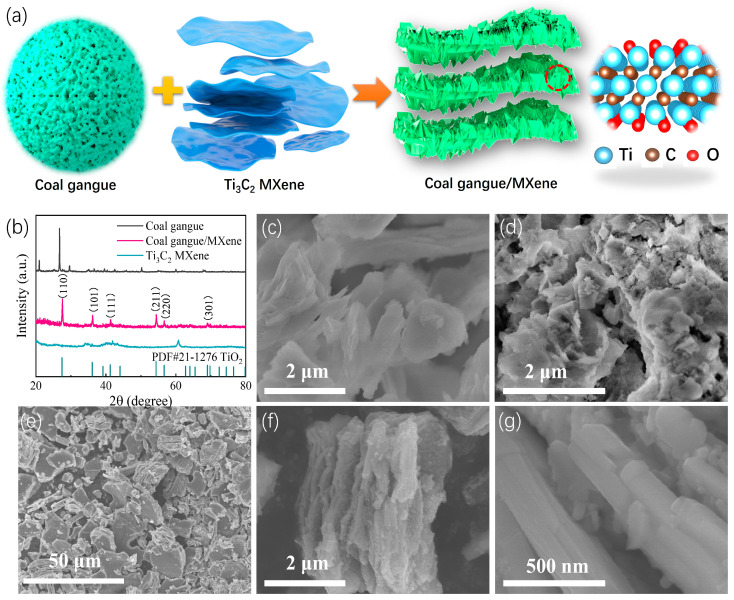
(**a**) Schematic description of the fabrication of coal gangue/MXene; (**b**) XRD pattern of all catalysts; SEM image of raw coal gangue (**c**), activated coal gangue (**d**), Ti_3_C_2_ MXene (**e**), and coal gangue/MXene (**f**,**g**).

The crystal structure and phase composition of the as-prepared catalysts were characterized using X-ray diffraction (XRD). As shown in Figure 1b, the apparent diffraction peaks, centered at 26.82°, 29.55°, and 42.52°, can be attributed to the existence of quartz, kaolinite, and hematite, respectively, in the coal gangue. This phenomenon indicates that the raw coal gangue is rich in aluminum-/silicon-based mineral components. However, the coal gangue/Ti_3_C_2_ MXene shows distinctive characteristic peaks of TiO_2_ (PDF#21-7206) and delivers no significant features of coal gangue, predicating that the coal gangue has an amorphous structure in the hybrid. The morphologic differences of all catalysts are measured through scanning electron microscopy (SEM) (Figure 1c–g). The raw coal gangue consists of irregular large particles, which is not conducive to the rapid diffusion of ions or the sufficient deposition of discharge products. After acid leaching activation, the coal gangue exhibited an unconsolidated colloidal structure with a smaller scale. The unique near-nanostructure form of the activated coal gangue could reduce the average free path of the charge and split the energy levels, thus exhibiting high electrochemical activity. Due to the in situ removal of impurities, a large number of pores were exposed on the surface of the activated coal gangue. In addition, monoclinic Ti_3_C_2_ MXene shows a multilayered, graphene-like structure (see Figure 1d). Following the composite treatment, the graphene-like sheets of Ti_3_C_2_ MXene were seen to be decorated with coal gangue nanoparticles (Figure 1f,g). The specific porous layered morphology of the coal gangue/Ti_3_C_2_ MXene hybrid is conducive to the provision of fast ion diffusion channels and an abundant storage area for discharge products in LOBs. Moreover, the unique composite structure can significantly enhance the synergistic effect between coal gangue and Ti_3_C_2_ MXene, leading to an enhanced electrocatalytic activity and the stability of the hybrid.

**Figure 2:**
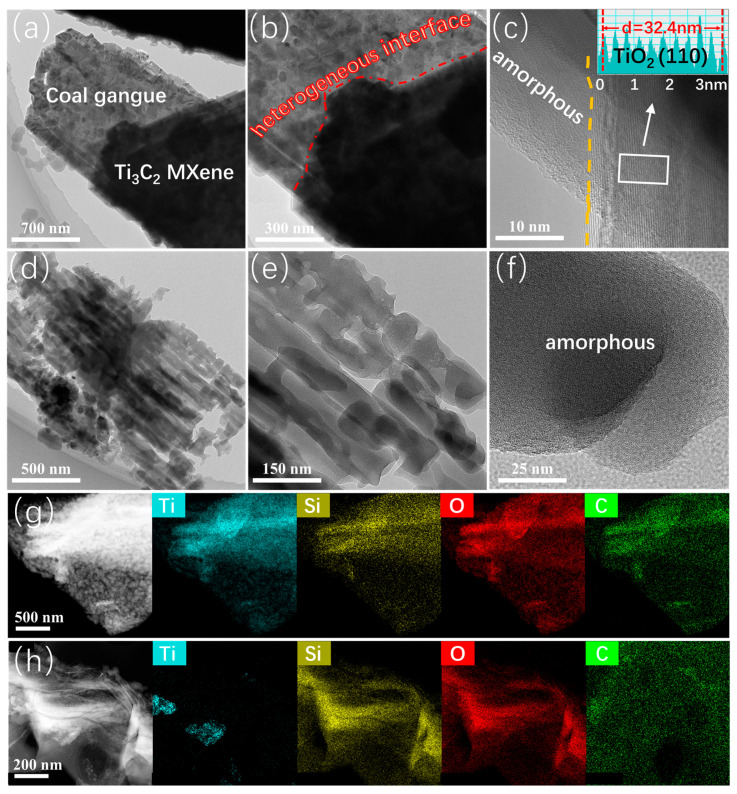
TEM image (**a**,**b**) HR-TEM image (**c**) of coal gangue/Ti_3_C_2_ MXene; TEM image (**d**,**e**) HR-TEM image (**f**) of coal gangue/Ti_3_C_2_ MXene; EDS mapping images of coal gangue/Ti_3_C_2_ MXene (**g**,**h**).

The deep-seated heterostructure of the coal gangue@Ti_3_C_2_ MXene hybrid was tested through a transmission electron microscope (TEM) analysis. As shown in Figure 2a, the coal gangue in the coal gangue@Ti_3_C_2_ MXene hybrid is composed of stacked nanoparticles. At the same time, there is an obvious irregular layer, with a thickness of 80 nm, that was generated on the surface of the coal gangue@Ti_3_C_2_ MXene hybrid; this could be ascribed to the self-assembled transitional metal oxide protective layer. More specifically, there is a heterogeneous interface between the coal gangue and the MXene; this can be seen in Figure 2b. This suggests the presence of an in situ-stable interface bridging and thereby an enhanced synergistic enhancement effect in the hybrid. Meanwhile, the coal gangue in the coal gangue@Ti_3_C_2_ MXene hybrid exhibits a special multi-nanoparticle stacking structure; this could be attributed to the influence of the activation of the acid and the alkali. Combined with the previous SEM picture, the unique nanoparticle-assembled colloidal structure could guarantee sufficient surface exposure of the active sites in the activated coal gangue. In short, the acid/alkali activation reaction and the following hydrothermal reaction could effectively modify the surface morphology of the coal gangue, resulting in the enhancement of electrocatalytic activity in LOBs. The nano effect among the coal gangue particles could facilitate charge diffusion and the presence of a lower ion transport barrier during the ORR/OER process of the LOBs. The excess holes existing between the coal gangue nanoparticles could be significant in promoting the complete immersion of the electrolyte and providing an adequate space for the deposition of solid discharge products. The high-resolution TEM image (Figure 2c) shows that the coal gangue@Ti_3_C_2_ MXene hybrid has amorphous/crystalline structures. To be specific, no obvious lattice fringes could be seen in the coal gangue, indicating that the formation of the amorphous structure came about following the activation of the coal gangue. It is easier to generate defect sites on the amorphous structure, and most of these defects can become active sites for the performance of ORR/OERs in LOBs. Furthermore, the characteristic lattice spacing measures about 0.324 nm in the crystalline region, indexed to the (110) planes for TiO_2_. Figure 2d shows the detailed microstructure of Ti_3_C_2_ MXene in the coal gangue@Ti_3_C_2_ MXene hybrid. Ti_3_C_2_ MXene displays a nanoscale, multilayered core–shell structure. Additionally, the outer shell of Ti_3_C_2_ MXene is a noncrystalline material, which could be attributed to formation of the protective coal gangue layer. The noncrystalline protective layer could take on a major dual role as an electrochemical catalytic site and a storage/decomposition area for the discharge products in LOBs. The atomic-resolution HAADF images of the coal gangue@Ti_3_C_2_ MXene hybrid shown in Figure 2g,h reveal that the hybrid is made up of Ti, Si, O, and C. Interestingly, there are plenty of Si atoms in the multilayered, sheet-like structure of the coal gangue@Ti_3_C_2_ MXene hybrid, which further confirms the existence of a silicon-based protective layer, derived from the coal gangue, on the surface of Ti_3_C_2_ MXene. In addition, the distribution of the oxygen elements is similar to that of silicon, suggesting the formation of silicon oxide sourced from coal gangue. Otherwise, the extensive spread of C among the coal gangue@Ti_3_C_2_ MXene hybrid proves the existence of a silicon carbide protective layer, which is in situ-coated on the surface of Ti_3_C_2_ MXene. In general, the coal gangue/Ti_3_C_2_ MXene hybrid was prepared well and delivers a unique graphene-like structure that is loaded with nanoparticles. The coal gangue/Ti_3_C_2_ MXene hybrid has a tailored amorphous/crystalline heterostructure, active TiO_2_ termination, and a stable SiC_x_ protective layer; therefore, it has better electrochemical catalytic activity.

**Figure 3:**
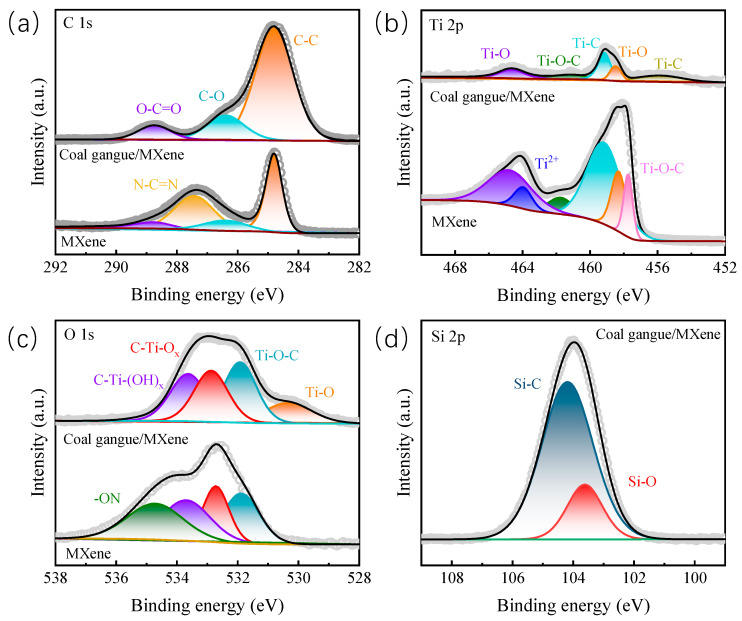
High-resolution C 1s (**a**), Ti 2p (**b**), O 1s (**c**), Si 2p (**d**) spectra for coal gangue/Ti_3_C_2_ MXene and Ti_3_C_2_ MXene.

X-ray photoelectron spectroscopy was adopted to further analyze the element composition and chemical state of the catalysts. As shown in Figure 3a, the characteristic peak located at 287.47 eV in Ti_3_C_2_ MXene could be assigned to N-C=N, which disappears in the coal gangue/Ti_3_C_2_ MXene hybrid [30]. The existence of coal gangue could prevent the nitrogen-based impurities forming on the surface of Ti_3_C_2_ MXene. For the Ti 2p spectra of Ti_3_C_2_ MXene (Figure 3b), the significant curve could be divided into six peaks, corresponding to Ti-O (464.89 and 458.33 eV), Ti^2+^ (464.00 eV), Ti-O-C (461.81 and 457.75 eV), and Ti-C (459.32 eV), respectively. By comparison, the coal gangue/Ti_3_C_2_ MXene hybrid delivers more obvious characteristic peaks of Ti-O, indicating the enhanced content of titanium oxide with higher catalytic activity. The O 1s spectra of Ti_3_C_2_ MXene, shown in Figure 3c, can deconvolute into four characteristic peaks. The distinct fitted peaks at 534.75, 533.72, 532.71, and 531.89 eV can be assigned to the presence of -ON, C-Ti-(OH)_x_, C-Ti-O_x_, and Ti-O-C, respectively [31]. Notably, the newly emerged feature peak of Ti-O in the coal gangue/Ti_3_C_2_ MXene hybrid further confirms the formation of titanium oxide on the surface of Ti_3_C_2_ MXene. In addition, there is no obvious peak of -ON in the coal gangue/Ti_3_C_2_ MXene hybrid, suggesting that coal gangue could promote the decomposition of nitrogen-based impurities and thereby raise the electrocatalytic performance of Ti_3_C_2_ MXene. The Si 2p spectra of the coal gangue/Ti_3_C_2_ MXene hybrid could deconvolute into two peaks at 104.19 and 103.62 eV; these can be attributed to the Si-C and Si-O bonds, respectively. The higher content of Si-C gives further evidence to the makeup of the SiCx active layer, which is consistent with the previous TEM mapping results. Inspired by the lack of characteristic peaks of silicon-based crystals in the XRD pattern, the in situ-prepared SiCx active layer has an amorphous structure in the coal gangue@Ti_3_C_2_ MXene hybrid. In general, combined with the previous TEM analysis, the TiO_2_/SiC_x_ active layer with the amorphous/crystalline heterostructure is in situ-formed on the coal gangue/Ti_3_C_2_ MXene electrocatalyst. The amorphous SiC_x_ protective layer could inhibit the nucleophilic aggregation of superoxide ions and thereby enhance the structural stability of the coal gangue/Ti_3_C_2_ MXene hybrid. In addition, the TiO_2_ catalytic units could act as the active sites in optimizing the ion diffusion pathway and regulating the adsorption/desorption path of solid products gathered on the cathode surface.

### 3.2. Aprotic Li-O_2_ Battery Performance

**Figure 4:**
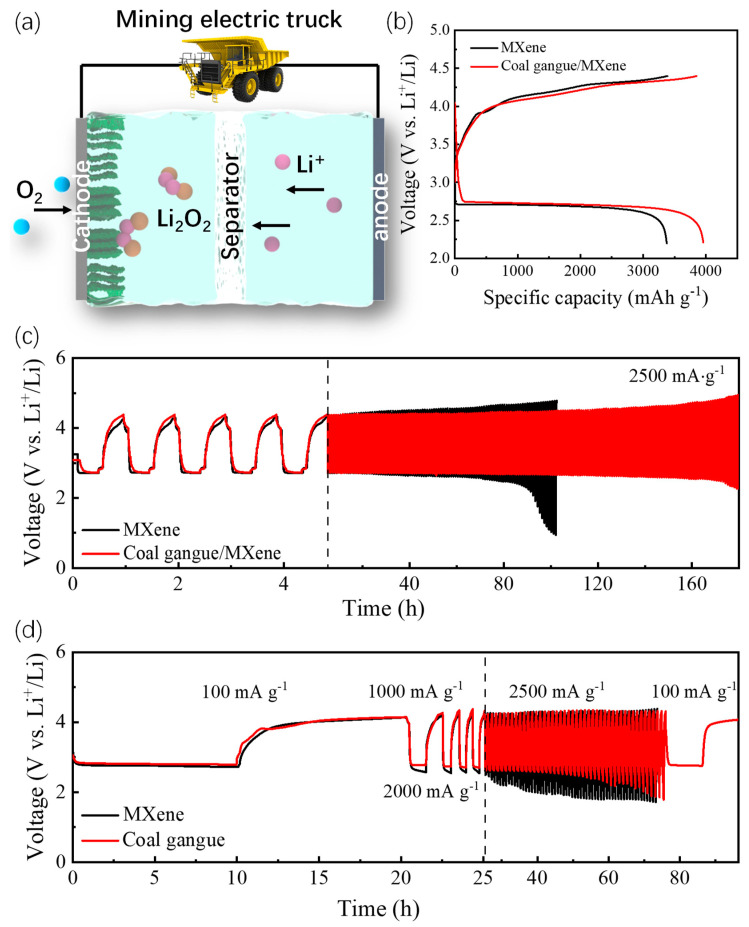
(**a**) Reaction mechanism of coal gangue/MXene-based LOBs. (**b**) Initial full discharge/charge curves of as-prepared catalyst-based LOBs at 2500 mA g^−1^; (**c**) cycling stability of as-prepared catalyst-based LOBs at 2500 mA g^−1^; (**d**) rate performances of as-prepared catalyst-based LOBs.

The electrocatalytic performances of the as-prepared catalysts are tested using 2032-coin type LOBs. The overall reaction mechanism of typical LOBs is graphically described in Figure 4a: the dissolved atmospheric O_2_ tends to react with adsorbed Li^+^, diffused from the lithium anode to form a solid Li_2_O_2_ product on the cathode at discharging. This could be reversibly broken down into O_2_ and Li^+^ after charging. Fundamentally, the cathode catalyst plays a key role in reducing the electrochemical reaction barriers of LOBs. At first, we applied the coal gangue/Ti_3_C_2_ MXene cathode catalyst to the LOBs with the motivation of improving the electrochemical performance, including the cycle stability, the rate, etc. As shown in Figure 4b, the coal gangue/Ti_3_C_2_ MXene hybrid represents a considerably bigger discharge-specific capacity of 3959 mAh g^−1^ at 2500 mA g^−1^. In addition, the coal gangue/Ti_3_C_2_ MXene hybrid delivers a slight difference in the discharge/charge voltage platform compared to that of the Ti_3_C_2_ MXene, indicating that the Ti_3_C_2_ MXene is the main active material in facilitating the kinetics of oxygen redox; meanwhile, coal gangue could optimize the electrochemical stability of Ti_3_C_2_ MXene. Furthermore, the coal gangue/Ti_3_C_2_ MXene-based LOBs could maintain a stable discharge/charge cycling for up to 180 h (about 175 cycles), as shown in Figure 4c). This is significantly superior to that of Ti_3_C_2_ MXene (~90 h). Additionally, despite the similar discharge/charge overpotential (1.61 V) observed in the early cycles, the coal gangue/Ti_3_C_2_ MXene hybrid shows a smaller voltage polarization of ~1.72 V to that of Ti_3_C_2_ MXene after about 40 h of cycling; this indicates that the collaborative enhancement effect derived from the coal gangue nanoparticles could efficiently modify the reaction kinetics and catalytic stability of the hybrid catalyst. The coal gangue/Ti_3_C_2_ MXene-based LOBs demonstrated highly consistent charging and discharging curves at different rates, as shown in Figure 4d. Specifically, the coal gangue/Ti_3_C_2_ MXene hybrid delivers stable voltage polarization from 1.31 V at 100 mA g^−1^ to 1.58 V at 2500 mA g^−1^. Moreover, the coal gangue/Ti_3_C_2_ MXene hybrid could sustain firmly for 50 h at the ultra-high rate of 2500 mA g^−1^. Importantly, the coal gangue/Ti_3_C_2_ MXene hybrid could maintain a homologous discharge/charge overpotential (~1.38 V) when the conducted current density returns to 100 mA g^−1^. Coal gangue, with an amorphous SiC_X_ protective layer as the stable porous substrate, could effectively enhance structural stability and provide an abundant area for the deposition of solid products. Moreover, the optimal in situ formation of the TiO_2_ active sites and the SiC_X_ protective layer could regulate the micro-chemical constitution and enhance the synergistic effect between coal gangue and Ti_3_C_2_ MXene. Compared with Ti_3_C_2_ MXene, coal gangue/Ti_3_C_2_ MXene delivers an enhanced electrochemical performance and a better cyclic life. 

**Figure 5:**
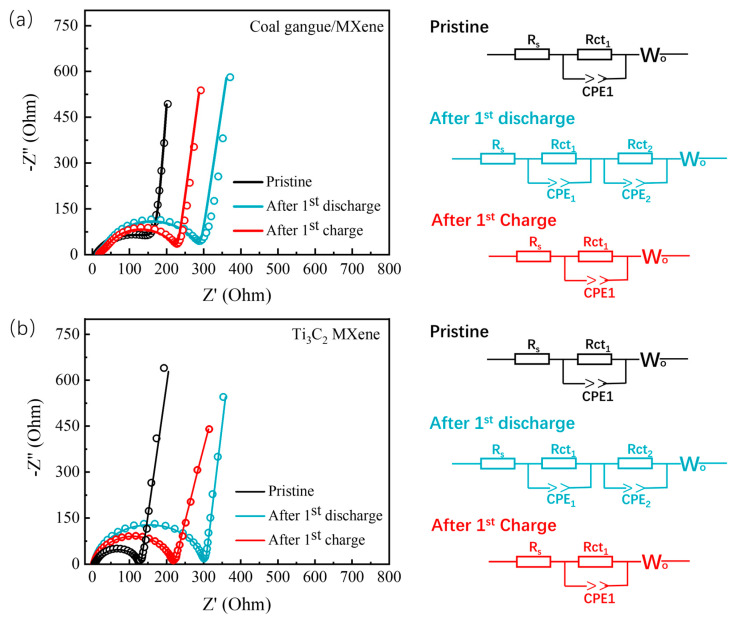
(**a**) Original (dot) and simulation (line) Nyquist plots of coal gangue/Ti_3_C_2_ MXene-based LOBs and EIS corresponding analog circuit diagram (right); (**b**) original (dot) and simulation (line) Nyquist plots of Ti_3_C_2_ MXene-based LOBs and corresponding EIS analog circuit diagram (right).

Figure 5 shows the electrochemical impedance spectra (EIS) of the coal gangue/Ti_3_C_2_ MXene hybrid and Ti_3_C_2_ MXene at different statuses. The ohmic resistance (R_0_) for the coal gangue/Ti_3_C_2_ MXene-based cathode (11.70 Ω for pristine state) is slightly larger than that of the Ti_3_C_2_ MXene-based cathode (5.79 Ω for pristine state). In addition, the charge transfer resistances (Rct) of the coal gangue/Ti_3_C_2_ MXene hybrid and Ti_3_C_2_ MXene after the first discharge were found to be 225.57 and 220.80 Ω, respectively. The larger Rct in the coal gangue/Ti_3_C_2_ MXene hybrid could be ascribed to the lower electric conductivity of the coal gangue. As shown in the EIS corresponding analog circuit diagram, CPE_2_ and Rct_2_ appeared after the first discharging point in both the coal gangue/Ti_3_C_2_ MXene hybrid and the Ti_3_C_2_ MXene; this indicates that a new interface was produced by the deposition of the discharge products. Furthermore, the Rct of the coal gangue/Ti_3_C_2_ MXene hybrid can recover to a lower numerical value after the following charging process, indicating the excellent catalytic properties of the coal gangue/Ti_3_C_2_ MXene hybrid in promoting the decomposition dynamics of Li_2_O_2_. In detail, the in situ-formed TiO_2_/SiC_X_ catalytic units could optimize the structural stability and expose a large number of catalytically active sites at ultra-high rates, thereby exhibiting excellent low-impedance characteristics.

## 4. Conclusions

In summary, the coal gangue/Ti_3_C_2_ MXene hybrid with TiO_2_/SiC_X_ catalytic-integrated units was fabricated and successfully applied as an electrocatalyst for LOBs. The LOBs with the coal gangue/Ti_3_C_2_ MXene cathode catalyst delivered a considerable long-term cycling lifespan of 180 h (up to 175 cycles) with a stable voltage polarization of 1.72 V at 2500 mA g^−1^. Furthermore, the coal gangue/Ti_3_C_2_ MXene hybrid exhibited an amazing rate performance and a subsequent self-healing feature. The excellent electrochemical performances could be attributed to three aspects: (1) The tailored amorphous/crystalline heterostructure of the whole hybrid could optimize the ion diffusion rate and expose a large number of catalytic active sites. (2) The multilayered, graphene-like morphology of Ti_3_C_2_ MXene makes significant contributions in improving the reaction kinetics and product storage of LOBs. (3) The in situ-formed TiO_2_/SiC_X_ catalytic units could regulate the micro-chemical constitution and enhance the synergistic effect between coal gangue and Ti_3_C_2_ MXene; in turn, this improves the overall stability of the whole hybrid. This work could pave the way for high-value-added applications of coal gangue in high-rate Li-O_2_ batteries.

## Data Availability

Data are contained within the article.
